# Digital Mirror Therapy and Action Observation Therapy for Chronic Stroke: A Pilot Randomized Controlled Trial

**DOI:** 10.1155/oti/8741362

**Published:** 2025-03-20

**Authors:** Yu-Wei Hsieh, Meng-Ta Lee, Ya-Ching Hsu, Kai-Yu Wu, Chih-Chi Chen

**Affiliations:** ^1^Department of Occupational Therapy and Graduate Institute of Behavioral Sciences, College of Medicine, Chang Gung University, Taoyuan, Taiwan; ^2^Department of Physical Medicine and Rehabilitation, Chang Gung Memorial Hospital at Linkou, Taoyuan, Taiwan; ^3^School of Medicine, College of Medicine, Chang Gung University, Taoyuan, Taiwan

**Keywords:** action observation, chronic stroke, digital mirror therapy, upper limb rehabilitation

## Abstract

**Objective:** This study is aimed at testing the immediate and retained treatment efficacies of digital mirror therapy (DMT) and digital action observation therapy (DAOT) on clinical outcomes in chronic stroke patients, in comparison with dose-matched, active control rehabilitation (CR).

**Methods:** Nineteen patients were randomly assigned to the DMT, DAOT, or CR for 3 weeks. Outcome measures, including the Fugl-Meyer Assessment of the Upper Extremity (FMA-UE), Chedoke Arm and Hand Activity Inventory (CAHAI), Revised Nottingham Sensory Assessment, Motor Activity Log (MAL), and visual analogue scale of the EQ-5D-5L, were conducted at preintervention (T0), postintervention (T1), and 1-month follow-up (T2).

**Results:** There were no significant differences among the three groups on the outcomes at different time points. However, in the DMT group, scores were significantly higher on the FMA-UE and the CAHAI at T2 than at T0; in the DAOT group, those on the FMA-UE and the quality of movement subscale of the MAL were significantly higher at T2 than at T0. In the CR group, scores on the FMA-UE and the CAHAI were significantly higher at T1 than at T0.

**Conclusions:** Both DMT and DAOT had retained treatment effects on motor function. DMT and DAOT might be feasible as alternative intervention strategies for chronic stroke patients.

**Trial Registration:** ClinicalTrials.gov identifier: NCT04441190

## 1. Introduction

Stroke still prevails as the second leading cause of death and the third leading cause of overall disease burden (as measured by disability-adjusted life-years, combining years lived with disability and years of life lost) throughout the world [1]. From 2010 to 2019, its global prevalence, incidence, deaths, and disability-adjusted life-years increased by 24.7%, 27.1%, 12.2%, and 9.6%, respectively [2]. As the global population rapidly ages in the coming years, the absolute number and incidence rates of stroke cases, particularly in the elderly, are likely to grow dramatically [3]. One of the most common problems in stroke survivors is upper limb impairments, including difficulty in movement and coordination of the arms, hands, and fingers, and such difficulty can lead to the inability to perform daily activities [4]. Over 50% of individuals with upper limb impairments following stroke have troubles in motor and daily functions lasting many months and years, so they are considered chronically disabled [5]. Because of the significant impact of stroke disability on disease burden and health expenditures, attention should be paid to providing effective and specific interventions with multifunctional and accessible rehabilitation facilities for upper limb motor rehabilitation.

In the recent past, mirror therapy (MT) and action observation therapy (AOT), both based on the motor priming paradigm, have become promising and effective interventions for improving the upper limb motor function of stroke patients [6]. During MT, patients are asked to perform motor movements concurrently while observing a mirror placed in front of the patient's midsagittal plane, in which the visually illusory reflection of the intact normal upper limb appears as if it were the paralyzed one [7]. During AOT, patients are asked to observe carefully motor actions from the video clips in the action observation phase and then actually perform the observed motor actions to the best of their ability in the action execution phase [8]. Previous meta-analyses have reported that, compared to control interventions, significant medium effect sizes for upper limb motor impairment and function [9, 10], as well as activities of daily living [9], favored MT; likewise, significant small-to-moderate effect sizes for upper limb motor impairment and function [11, 12] and moderate-to-large effect sizes for activities of daily living [11] favored AOT. With the benefit of recent advances in digital imaging technology, we have developed a digital stroke rehabilitation system, which incorporates the two interventions of digital mirror therapy (DMT) and digital action observation therapy (DAOT) into the same software and hardware. This system has been validated for good usability and user experiences from the perspective of both therapists and stroke patients [13].

During the DMT in this system, patients are asked to perform movements of the intact normal upper limb, in which those movements are captured by a webcam and then instantly transformed into mirror images of the movements presented on the screen over the paralyzed upper limb, and patients can simultaneously observe the mirror images of the movements from the front, not from the side [13, 14]. Digitalized MT has some advantages over classic MT, which is applied by using a plain mirror or mirror box, as follows: (a) minimization of neck tension, tilting of head and trunk, asymmetry of body alignment, and weight shifting and leaning toward the intact normal side [13, 15]; (b) the possibility of performing asymmetrical upper limb movements [16] or increasing the diversity of the range of motion exercise and daily tasks [13, 17]; (c) lifelike and persuasive visually illusory reflections on the screen [13, 15, 18]; and (d) the possibility of bilateral mirror visual feedback superimposed on both the intact normal and paralyzed upper limbs [13, 15], not merely a unilateral mirror visual feedback image presented by a mirror.

In addition, during the DAOT of this system, similarly to general AOT, the procedure consists of first carefully observing movements in video clips in the action observation phase and then physically performing the observed movements in the action execution phase [13]. However, the DAOT of this system provides two kinds of videos, namely, prerecorded and self-recorded videos, to meet the various needs of users. The prerecorded videos of the DAOT in the system include the categories of active range of motion (AROM), reaching movement, object manipulation, and upper limb functional tasks so as to provide diverse movements or actions as treatment options. Furthermore, either the patients or other individuals (e.g., therapists and caregivers) can perform the movements with their intact normal upper limbs; such movements can be captured by the webcam and further recorded and stored as self-recorded videos for tailoring interventions to meet patients' individualized needs and therapeutic goals [13].

This novel digital stroke rehabilitation system, which addresses the disadvantages of classic MT and AOT and integrates those two promising and effective interventions into one system in a digital way (i.e., DMT and DAOT), achieves a more eclectic approach for clinical practice. With digitalization, this system offers several specific benefits: (a) mirror visual feedback can be observed from the front rather than the conventional side view, enhancing practicality. (b) It allows for the creation of various mirror visual feedback conditions in combination with different manual exercises, enabling diverse training modes. (c) It provides the flexibility to create self-recorded videos for diverse functional tasks within a real-life environment. (d) It enables the easy exchange between DMT and DAOT for treatment [13]. These features allow stroke patients to receive therapy in ergonomically improved positions and with closer alignment to their daily needs. Moreover, therapists can select the most suitable therapeutic mode or combine different training modes, offering more flexible, meaningful, and engaging treatments for patients. However, some limitations of incorporating digitization into this system are as follows: (a) The system requires a certain level of familiarity with technology, which could pose challenges for patients less experienced with digital tools. (b) The initial setup of hardware and software may necessitate additional resources and training.

The treatment effects of DMT and DAOT using this system in stroke patients are unknown and need to be examined for further clinical use. This pilot study is aimed at testing the immediate and retained treatment efficacies of DMT and DAOT on different aspects of rehabilitation outcomes in chronic stroke patients and to compare these efficacies with those of dose-matched, active conventional occupational therapy.

## 2. Methods

### 2.1. Participants

Participants were recruited from physical medicine and rehabilitation departments of three hospitals in northern Taiwan. The inclusion criteria of the participants were as follows: (a) diagnosis of unilateral stroke, (b) stroke onset more than 6 months before participation, (c) age of 20–80 years; (d) baseline Fugl-Meyer Assessment of the Upper Extremity (FMA-UE) score of 20–60 [19], and (e) ability to follow the study instructions (a score of ≥ 21 on the Mini-Mental State Examination, Second Edition: Standard Version (MMSE-2:SV)) [20]. Furthermore, eligible participants were excluded for the following reasons: (a) diagnosis of global or receptive aphasia, (b) severe neglect (mean deviations ≥ 1.5 in. on the 8-in. horizontal line on the line bisection subtest of the Behavioral Inattention Test), or (c) major medical problems or comorbidities that could interfere with upper limb usage or cause severe pain.

This study was approved by the Institutional Review Board of the Chang Gung Memorial Hospital (201901885A3), the Taipei Hospital, Ministry of Health and Welfare (TH-IRB-0020–0004), and the Taoyuan General Hospital, Ministry of Health and Welfare (TYGH109043), and written informed consent was obtained from each participant.

### 2.2. Study Design and Procedure

This pilot study was a three-arm, single-blind, randomized-controlled trial. All participants received the study intervention, which comprised a total of 15 training sessions (60 min per session) for 3–4 weeks apart from routine rehabilitation (e.g., occupational, physical, or speech therapy). During the study period, well-trained research staff with an occupational therapy license provided individually face-to-face study intervention to the participants with verbal instructions, assistance, and feedback as appropriate in the therapy room. Besides, the same blinded assessor administered the outcome measures to the participants at preintervention (T0), at postintervention (T1), and at the 1-month follow-up after the intervention (T2).

After the baseline evaluation, the participants were randomly allocated to one of the three arms of treatment groups in a 1:1:1 ratio according to the stratified random sampling method (i.e., FMA-UE score: 20–40 (moderate to severe) vs. 40–60 (mild to moderate) [19] and lesion side (right vs. left)). The random allocation was conducted online on a freely available website (https://www.randomizer.org/). For allocation concealment, a research assistant was independently responsible for the randomization procedure and group allocation.

### 2.3. Intervention

The two experimental groups of DMT and DAOT received the intervention protocols delivered by the digital stroke rehabilitation system [13], whereas the dose-matched control rehabilitation (CR) group received conventional occupational therapy as an upper limb rehabilitation intervention.

#### 2.3.1. DMT

**Figure 1:**
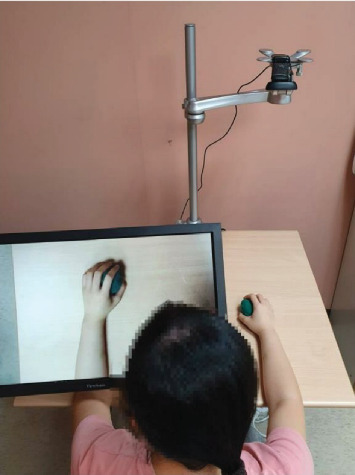
Demonstration of digital mirror therapy.

During DMT, the movements of the patient's nonaffected hand (e.g., right hand) were recorded, instantly transformed into mirror images of the affected hand's movements (e.g., left hand), and presented on the screen (Figure 1). The images of the nonaffected arm and hand were captured by the webcam, transformed by the software, and superimposed on the affected arm and hand. At the same time, the patients were required to imagine that the movements on the screen were performed by their affected arm and hand. For the AROM exercise period (15 min), the patients observed transformed AROM exercise on the screen, imagined it as the movements of the affected arm and hand, and simultaneously moved the affected arm and hand as much as possible. The AROM exercises included elbow, forearm, wrist, and finger movements. For the reaching movement or object manipulation period (20 min), based on each patient's level of motor function, the patients were asked to perform one to two reaching movements/object manipulation tasks per session. For the upper limb functional task period (25 min), the patients executed one to two functional tasks per session, beginning with easy tasks and gradually performing functional tasks of higher complexity. Examples of upper limb functional tasks are folding a towel, squeezing a sponge, flipping a card, and wiping a table.

#### 2.3.2. DAOT

**Figure 2:**
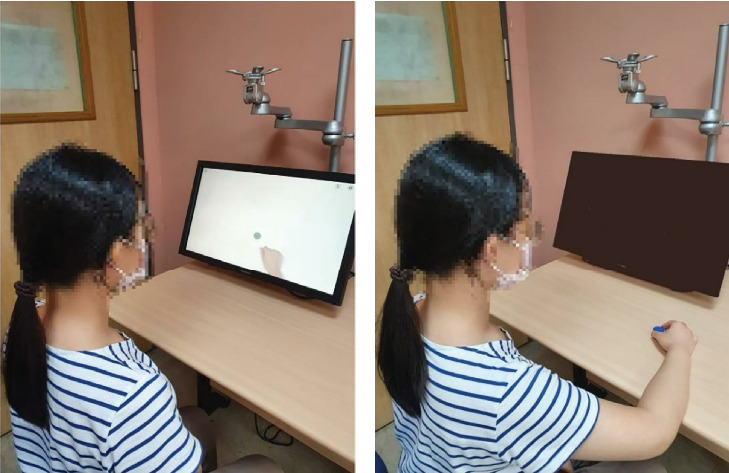
Demonstration of digital action observation therapy: (a) action observation phase and (b) action execution phase.

For the DAOT group, both prerecorded videos in the system (about 120 video clips) and self-recorded videos could be selected and used. The two common phases implemented during DAOT were the action observation and the action execution (i.e., physical practice) phases (Figure 2). The patients observed one video clip of a movement or task for 2 min and then practiced executing the same action for 3 min, which could be repeated. For the AROM exercise period (15 min), video clips of shoulder, elbow, forearm, wrist, and finger movements could be selected. For the reaching movement or object manipulation period (20 min), the system offered about 50 video clips of reaching for objects of different sizes and weights at different heights and locations, as well as clips of in-hand manipulation, grasping and releasing objects, and transporting and turning objects. For the upper limb functional task period (25 min), the system contained about 50 video clips of tasks, such as buttoning up a shirt/blouse, folding a towel, using a cellphone, wiping a table, opening a bag, and cutting a cake.

#### 2.3.3. CR

The CR group received dose-matched, active conventional occupational therapy for upper limb training. As in the DMT and DAOT groups, the three common categories of motor actions and tasks, namely, (a) AROM exercises (15 min), (b) reaching movement or object manipulation (20 min), and (c) upper limb functional tasks (25 min), were conducted. The therapist adjusted the difficulty of movements or tasks according to the patient's levels of motor function as appropriate.

### 2.4. Outcome Measures

#### 2.4.1. Primary Outcomes

The upper extremity subscale of the Fugl-Meyer Assessment (FMA-UE) comprises 33 items for measuring the reflexes and movements of the shoulder/elbow/forearm, wrist, and hand, as well as coordination/speed [19]. Its total score range is 0–66, with a higher score indicating better upper limb motor function.

The Chedoke Arm and Hand Activity Inventory (CAHAI) is a reliable and validated performance-based test, consisting of 13 real-life functional tasks, for assessing the functional ability of stroke patients to perform daily activities by using their affected upper limbs [21]. Its total score range is 13–91, with a higher score indicating greater recovery of arm and hand function.

#### 2.4.2. Secondary Outcomes

The Revised Nottingham Sensory Assessment (rNSA) is a standardized measure with sound psychometric properties for assessing sensory function in stroke patients [22]. For its tactile sensation subscale, each region of the body is tested for light touch, temperature, pinprick, pressure, tactile location, and bilateral simultaneous touch and scored from 0 (absent) to 2 (normal). For its proprioception subscale, each region of the body is scored on a range of 0 (absent) to 3 (joint position sense). For its stereognosis subscale, each object is scored from 0 (absent) to 2 (normal). A higher score indicates better somatosensory function.

The Motor Activity Log (MAL) is a semistructured questionnaire with good psychometric properties for assessing the level of using the affected upper limb when performing 30 daily tasks, and performance is scored in two aspects: amount of use (AOU) and quality of movement (QOM) [23]. The total mean score range of each aspect is 0–5, with a higher mean score indicating better daily function.

The visual analogue scale (VAS) of the EQ-5D-5L is a quantitative, self-rated measure on a 20-cm vertical line for assessing health status with a score range of 0–100 from the worst (at the bottom) to the best (at the top) imaginable health state [24]. A higher score indicates a better overall current health state.

### 2.5. Statistical Analysis

IBM SPSS 22.0 software was used to analyze the data. A two-sided *p* value of < 0.05 was noted as statistically significant. The nonparametric Kruskal–Wallis test was used to compare the demographic and baseline clinical characteristics among the DMT, DAOT, and CR groups. The intention-to-treat analysis with the last observation carried forward method was adopted to deal with missing data.

The nonparametric Friedman test was used to examine the within-group differences in the scores of outcomes among T0, T1, and T2. Kendall's *W* value was used to measure the effect size of the Friedman test, and *W* values of 0.1, 0.3, and 0.5 reflected small, medium, and large effect sizes, respectively. Furthermore, the nonparametric Kruskal–Wallis test was used to examine the between-group differences in the change scores of the outcomes from T0 to T1 and from T0 to T2 among the three groups. The *ε*^2^ value was used to measure the effect size of the Kruskal–Wallis test; *ε*^2^ values of 0.01, 0.06, and 0.14 indicated small, medium, and large effect sizes, respectively. If the Friedman test or Kruskal–Wallis test was significant, Dunn's test with Bonferroni correction was performed for post hoc multiple pairwise comparisons. For the pairwise comparisons, a *p* value < 0.017 was noted as statistically significant. Additionally, whether the mean change scores of the outcomes in each intervention group reached the minimal clinically important difference (MCID) was reported. For outcome measures with established MCID values, these values were employed; for those without, a value estimated to be approximately 10% of the range score [25] was employed. Thus, the MCID values of the FMA-UE [26], CAHAI [27], tactile sensation of the rNSA, proprioception and stereognosis subscales of the rNSA, AOU and QOM of the MAL [28], and VAS of the EQ-5D-5L [29] were 4.25, 5, 11, 3, 3, 0.5, and 9 points, respectively.

## 3. Results

### 3.1. Demographic and Baseline Clinical Characteristics

**Figure 3:**
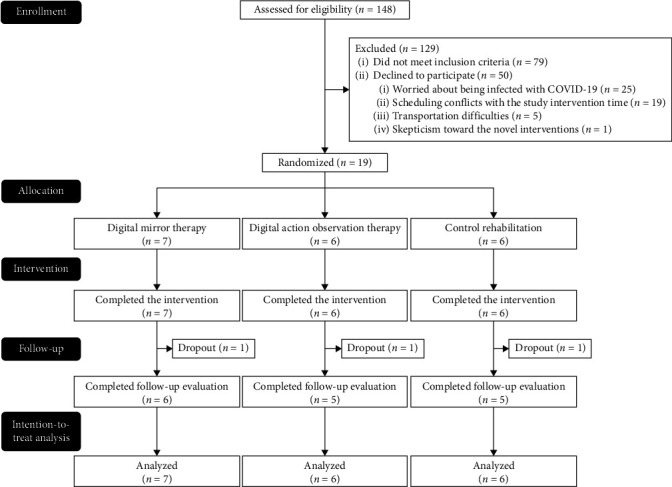
Flowchart of study participants.

During the COVID-19 pandemic, between August 2020 and May 2021, a total of 19 stroke patients were recruited and then randomized to one of the three interventions (Figure 3). The demographic and baseline clinical characteristics of the participants are presented in Table 1. There were no significant differences in the demographic and baseline clinical characteristics among the three groups. Additionally, all the participants complied with and completed the intervention protocols, except for the three patients who dropped out due to confirmed cases of COVID-19 or hospital shutdown. During the course of the study intervention, no adverse effect was observed.

### 3.2. Within-Group Changes

#### 3.2.1. Primary Outcomes

In the DMT group, there were significant differences among T0, T1, and T2 with large effect sizes on the scores of the FMA-UE (*p* = 0.002), as well as the CAHAI (*p* = 0.005). Furthermore, the post hoc pairwise comparisons showed that the scores on the FMA-UE (*p* = 0.001) and CAHAI (*p* = 0.006) were significantly greater at T2 than at T0 (Table 2). Besides, in the DAOT group, there were significant differences among T0, T1, and T2 with large effect size on the scores of the FMA-UE (*p* = 0.004). Furthermore, the post hoc pairwise comparisons showed that the scores on the FMA-UE (*p* = 0.001) were significantly greater at T2 than at T0 (Table 2). Additionally, in the CR group, there were significant differences among T0, T1, and T2 with large effect sizes on the scores of the FMA-UE (*p* = 0.019) and CAHAI (*p* = 0.006). Furthermore, the post hoc pairwise comparisons showed that the scores on the FMA-UE (*p* =0.014) and CAHAI (*p* = 0.002) were significantly greater at T1 than at T0 (Table 2).

#### 3.2.2. Secondary Outcomes

In the DMT group, there were significant differences among T0, T1, and T2 with large effect sizes on the VAS of the EQ-5D-5L (*p* = 0.018) (Table 3). Moreover, in the DAOT group, there were significant differences among T0, T1, and T2 with large effect sizes on the tactile sensation (*p* = 0.030) and proprioception (*p* = 0.037) subscales of the rNSA and the QOM of the MAL (*p* = 0.015). Furthermore, the post hoc pairwise comparisons showed that the scores on the QOM of the MAL (*p* = 0.006) were significantly greater at T2 than at T0 (Table 3). However, in the CR group, there was no significant difference among T0, T1, and T2 on each outcome (Table 3).

### 3.3. Between-Group Comparisons

There were nonsignificant between-group differences, but different magnitudes of effect sizes on each outcome among the DMT, DAOT, and CR groups were found from T0 to T1 and from T0 to T2 (Table 4).

#### 3.3.1. Primary Outcomes

From T0 to T1, small and medium effect sizes were found, respectively, in the change scores on the FMA-UE and CAHAI among the three groups. The mean changes of all three groups reached the MCID value of the FMA-UE and CAHAI. Besides, from T0 to T2, medium and small effect sizes were found, respectively, in the change scores on the FMA-UE and CAHAI among the three groups. The mean changes of all three groups also reached the MCID value of the FMA-UE and CAHAI.

#### 3.3.2. Secondary Outcomes

From T0 to T1, large effect sizes were found in the change scores on the stereognosis subscale of the rNSA, the AOU of the MAL, and the VAS of the EQ-5D-5L among the three groups. Medium effect sizes were found in the change scores of the tactile sensation and proprioception subscales of the rNSA among the three groups. The mean changes of the CR group reached the MCID value of the VAS of the EQ-5D-5L. Besides, from T0 to T2, large effect sizes were found in the change scores on the tactile sensation, proprioception, and stereognosis subscales of the rNSA among the three groups. Furthermore, medium effect sizes were found in the changes of the AOU of the MAL among the three groups. The mean changes of the CR group reached the MCID value of the two subscales of the MAL.

## 4. Discussion

This is the first study to test the immediate and retained treatment efficacies of DMT and DAOT delivered with a digital stroke rehabilitation system on different aspects of rehabilitation outcomes in chronic stroke patients. The results of this study revealed that in terms of within-group changes, in the DMT group, the scores of the FMA-UE and the CAHAI at 1-month follow-up after intervention were significantly greater than those at preintervention. In the DAOT group, the scores of the FMA-UE and the QOM of the MAL at 1-month follow-up after intervention were significantly greater than those at preintervention. However, in the CR group, the scores of the FMA-UE and the CAHAI at postintervention were significantly greater than those at preintervention. Our preliminary findings showed that the patients in the DMT and DAOT groups exhibited significant retained effects, respectively, on motor function and functional abilities of the affected upper limb, as well as on motor function and movement quality of the affected upper limb. However, the patients in the CR group merely gained significant immediate intervention effects on motor function and functional abilities of the affected upper limb.

In this pilot study, the results demonstrated that instead of immediate treatment efficacy, the DMT and DAOT groups demonstrated retained (i.e., 1-month follow-up after intervention) treatment efficacy on upper limb motor function, and the improvements in functional abilities and movement quality were, respectively, retained in the two groups. One possible explanation for this phenomenon would be that the small sample size of each group in this study led to a lack of sufficient statistical power. The mean changes of the FMA-UE scores in the DMT and DAOT groups were 5.86 and 4.83 from T0 to T1 and 7.86 and 6.83 from T0 to T2; all of which met or exceeded the MCID values of the FMA-UE in chronic stroke [26]. These results indicated that there might be a real immediate treatment effect after intervention (i.e., the improvements reached MCID), but statistical significance was not achieved because the DMT and DAOT groups had only seven and six patients, respectively [30]. Another possible reason might be that the total treatment dosage of the DMT and DAOT in this study was relatively lower than that of the MT and AOT in some previous studies. For example, in the study of Amasyali et al., which featured a total of 1800 min of MT, significant immediate and retained treatment effects on motor function, hand dexterity, and grip force were found, respectively, at the end of a 3-week intervention and at 3-month follow-up after intervention [31]. Furthermore, in the meta-analysis of Zeng et al., consisting of 12 articles, the total amount of MT time in each trial ranged from 400 to 1920 min [10]; in the meta-analysis of Zhang et al., consisting of seven trials, the total amount of AOT time in each trial ranged from 600 to 1620 min [12]. However, in our study, the total amount of the therapy time in the DMT and DAOT groups was only 900 min. Further research is needed to explore the optimal intervention dosages of the digital types of MT and AOT for stroke patients.

Not surprisingly, nonsignificant differences in the change scores on the outcomes were found in between-group comparisons among the three groups. One explanation for the findings of this study might be the dose-matched, active conventional occupational therapy applied in the CR (control) group. Our control group was provided the same three categories of motor actions and tasks, including AROM exercises, reaching movement or object manipulation, and upper limb functional tasks, as the DMT and DAOT groups were. The main difference in intervention between the CR and the other two groups was that the therapists implemented the techniques for upper limb training (e.g., neurodevelopmental, motor learning, and task-oriented approaches) without providing either videos or mirror illusions of movements for observation. Nevertheless, the CR group exhibited similar trends to the DMT and DAOT groups on most outcomes from T0 to T1 but not from T0 to T2. This difference may be explained by the use of motor imagery techniques in the two experimental groups, specifically through the actual implementation of this strategy in the DMT group and its application prior to subsequent motor practice (i.e., the action observation phase) in the DAOT group. The structured and repetitive use of motor imagery combined with physical practice not only provides opportunities to increase the number of repetitions but also improves upper limb function more effectively than physical exercise alone [6]. Additionally, as presented in Table 4, those nonsignificant results but with large effect sizes in the change scores of the outcomes from T0 to T1 and from T0 to T2 among the three groups implied the potentially practical significance. Further large-scale studies are suggested to evaluate the comparative treatment effects of DMT, DAOT, and dose-matched, active conventional occupational therapy in stroke rehabilitation.

In comparison with previous conventional MT and AOT studies, we found that conventional MT studies generally reported immediate treatment effects; however, the retention of these effects varied. Studies that reported significant immediate effects include Wu et al. [32], Michielsen et al. [33], Cacchio et al. [34], and Yavuzer et al. [35]. However, only Cacchio et al. [34] and Yavuzer et al. [35] demonstrated significant between-group differences favoring MT at follow-up, whereas Wu et al. [32] and Michielsen et al. [33] found that the improvements did not persist. For AOT, studies generally suggest that treatment effects can be retained. Studies reporting both immediate and follow-up effects include Sale et al. [36] and Franceschini et al. [37]. Additionally, Ertelt et al. [38] found significant immediate improvements in the AOT group, with no significant decline at follow-up. Our study showed that although there were no significant differences among the three groups at different time points, within-group comparisons revealed that the DMT and DAOT groups exhibited significantly retained effects on some outcomes. In contrast, the CR group showed significant immediate effects. Compared to conventional MT and AOT interventions, our findings indicate that digital MT and AOT may provide comparable or potentially more sustained treatment effects. The differences in retention observed across studies may be influenced by variations in intervention protocols, patient populations, and outcome measures. Further research is needed to explore the long-term efficacy of digital interventions and their potential advantages over conventional approaches.

The key contribution of this pilot study is the translation of laboratory research on this digital stroke rehabilitation system into clinical use. With the validation of its treatment efficacy, both DMT and DAOT might be considered as feasible and useful interventions in stroke rehabilitation in day-to-day clinical practice.

Some limitations of this study should be noted. First, this pilot study did not have a sufficiently large sample size to achieve statistical significance for the immediate and retained treatment effects on different aspects of outcomes among the three groups. Under the limitation of the small sample size, the statistically significant findings still deserve our attention; however, the statistically nonsignificant findings should be viewed conservatively, especially those on the outcomes with large effect sizes. Further work with a larger sample is warranted to confirm the results of this pilot study. Second, the diversity in baseline clinical characteristics of stroke patients, such as hemorrhagic/ischemic stroke types, right/left side sites of brain lesions, and the location of stroke, may have increased the variability among the patients in this study, so the results must be interpreted with caution, especially in view of the small sample size. It is further suggested that the possibility of stroke type, brain lesion site, location of stroke, or severity of upper limb motor impairment of the patients affecting the treatment efficacy of this system delivering DMT and DAOT be investigated. Third, even with the same total amount of therapy time, the number of repetitions performed by study participants for each motor action and task category within the three groups may also affect treatment efficacy. Further research is recommended to report the number of repetitions, in addition to the total amount of treatment time, as a reference for intervention dosage.

## 5. Conclusion

In conclusion, both DMT and DAOT using a digital stroke rehabilitation system effected maintainable improvements on motor function and, respectively, on functional abilities and movement quality of the affected upper limb. In contrast, the dose-matched, active conventional occupational therapy provided immediate improvements on motor function and functional abilities of the affected upper limb. Based on the findings of this pilot study, DMT and DAOT might be feasible as alternative intervention strategies for improving the upper limb motor function of chronic stroke patients.

## Figures and Tables

**Table 1:** Demographic and baseline clinical characteristics.

**Characteristics**	**DMT** **(** **n** = 7**)**	**DAOT** **(** **n** = 6**)**	**CR** **(** **n** = 6**)**	**p** ** value**
Sex (male/female)	5/2	0/6	6/1	0.390
Age (years)	49.84 ± 7.70	47.74 ± 9.29	54.19 ± 17.65	0.249
Handedness (right/left)	7/0	6/0	5/1	0.338
Time since stroke onset (months)	14.29 ± 4.61	23.50 ± 18.81	30.17 ± 21.79	0.339
Lesion side (right/left)	2/5	3/3	3/3	0.674
Stroke type (hemorrhagic/ischemic)	5/2	2/4	3/3	0.406
Education (years)	12.14 ± 5.18	12.17 ± 2.23	12.67 ± 1.63	0.548
MMSE-2:SV score (0–30)	26.29 ± 2.43	27.83 ± 1.33	27.83 ± 2.93	0.320
FMA-UE score (0–66)	39.14 ± 11.57	37.00 ± 12.18	37.83 ± 15.82	0.913
CAHAI score (13–91)	40.14 ± 23.08	37.83 ± 17.15	34.17 ± 16.89	0.776
rNSA–tactile sensation score (0–108)	79.86 ± 34.50	80.50 ± 38.50	89.00 ± 22.40	0.770
rNSA–proprioception score (0–21)	17.71 ± 4.86	17.17 ± 4.79	17.83 ± 3.82	0.902
rNSA–stereognosis score (0–22)	15.43 ± 8.46	16.00 ± 6.81	14.00 ± 6.57	0.648
MAL–AOU score (0–5)	1.86 ± 1.38	1.72 ± 1.28	1.23 ± 1.04	0.680
MAL–QOM score (0–5)	1.72 ± 1.21	1.76 ± 1.25	1.31 ± 1.02	0.643
EQ-5D-5L–VAS score (0–100)	81.14 ± 14.17	66.67 ± 18.35	70.83 ± 19.34	0.355

*Note:* Values are presented as frequencies or mean ± standard deviation (SD).

Abbreviations: AOU, amount of use; CAHAI, Chedoke Arm and Hand Activity Inventory; CR, control rehabilitation group; DAOT, digital action observation therapy group; DMT, digital mirror therapy group; FMA-UE, Fugl-Meyer Assessment of the Upper Extremity; MAL, Motor Activity Log; MMSE-2:SV, Mini-Mental State Examination, 2nd Edition: Standard Version; QOM, quality of movement; rNSA, Revised Nottingham Sensory Assessment; VAS, visual analogue scale.

**Table 2:** Within-group changes of the scores on primary outcome measures at preintervention (T0), postintervention (T1), and 1-month follow-up after intervention (T2).

**Primary outcomes**	**Group**	**T0**	**T1**	**T2**	**p** ** value**	**W**	**Pairwise comparison**
FMA-UE (0–66)	DMT (*n* = 7)	39.14 ± 11.57	45.00 ± 14.38	47.00 ± 14.98	0.002⁣^∗^	0.93	T2 > T0
	DAOT (*n* = 6)	37.00 ± 12.18	41.83 ± 11.72	43.83 ± 12.97	0.004⁣^∗^	0.94	T2 > T0
	CR (*n* = 6)	37.83 ± 15.82	44.50 ± 12.79	42.67 ± 15.32	0.019⁣^∗^	0.66	T1 > T0

CAHAI (13–91)	DMT (*n* = 7)	40.14 ± 23.08	47.14 ± 26.42	50.29 ± 26.53	0.005⁣^∗^	0.76	T2 > T0
	DAOT (*n* = 6)	37.83 ± 17.15	45.50 ± 22.25	45.50 ± 20.45	0.337	0.18	
	CR (*n* = 6)	34.17 ± 16.89	44.83 ± 19.36	42.67 ± 21.31	0.006⁣^∗^	0.85	T1 > T0

*Note:* Values are presented as mean ± standard deviation (SD).

Abbreviations: CAHAI, Chedoke Arm and Hand Activity Inventory; CR, control rehabilitation group; DAOT, digital action observation therapy group; DMT, digital mirror therapy group; FMA-UE, Fugl-Meyer Assessment of the Upper Extremity.

⁣^∗^*p* < 0.05.

**Table 3:** Within-group changes of the scores on secondary outcome measures at preintervention (T0), postintervention (T1), and 1-month follow-up after intervention (T2).

**Secondary outcomes**	**Group**	**T0**	**T1**	**T2**	**p** ** value**	**W**	**Pairwise comparison**
rNSA–tactile sensation (0–108)	DMT (*n* = 7)	79.86 ± 34.50	83.29 ± 32.46	90.43 ± 23.36	0.056	0.41	
	DAOT (*n* = 6)	80.50 ± 38.50	84.67 ± 35.03	88.67 ± 29.31	0.030⁣^∗^	0.58	
	CR (*n* = 6)	94.60 ± 19.79^†^	98.20 ± 19.24^†^	98.60 ± 21.02^†^	0.867	0.03	

rNSA–proprioception (0–21)	DMT (*n* = 7)	17.71 ± 4.86	18.57 ± 4.47	17.86 ± 5.96	0.584	0.08	
	DAOT (*n* = 6)	17.17 ± 4.79	18.33 ± 3.62	18.67 ± 3.83	0.037⁣^∗^	0.55	
	CR (*n* = 6)	18.80 ± 3.35^†^	19.00 ± 3.39^†^	19.40 ± 1.95^†^	0.717	0.07	

rNSA–stereognosis (0–22)	DMT (*n* = 7)	15.43 ± 8.46	13.71 ± 7.70	14.57 ± 7.72	0.305	0.17	
	DAOT (*n* = 6)	16.00 ± 6.81	16.67 ± 5.47	17.00 ± 3.74	0.646	0.07	
	CR (*n* = 6)	14.00 ± 7.38^†^	15.20 ± 7.95^†^	16.00 ± 9.27^†^	0.368	0.20	

MAL–AOU (0–5)	DMT (*n* = 7)	1.86 ± 1.38	2.21 ± 1.43	2.18 ± 1.55	0.163	0.26	
	DAOT (*n* = 6)	1.72 ± 1.28	1.81 ± 1.38	2.07 ± 1.39	0.311	0.19	
	CR (*n* = 6)	1.23 ± 1.04	1.51 ± 1.12	1.99 ± 1.28	0.074	0.43	

MAL–QOM (0–5)	DMT (*n* = 7)	1.72 ± 1.21	2.02 ± 1.21	2.03 ± 1.41	0.482	0.10	
	DAOT (*n* = 6)	1.76 ± 1.25	1.95 ± 1.31	2.22 ± 1.32	0.015⁣^∗^	0.70	T2 > T0
	CR (*n* = 6)	1.31 ± 1.02	1.45 ± 0.99	1.83 ± 1.27	0.074	0.43	

EQ-5D-5L–VAS (0–100)	DMT (*n* = 7)	81.14 ± 14.17	87.14 ± 11.41	89.86 ± 11.57	0.018⁣^∗^	0.57	
	DAOT (*n* = 6)	66.67 ± 18.35	70.00 ± 13.78	74.50 ± 16.29	0.465	0.13	
	CR (*n* = 6)	70.83 ± 19.34	83.33 ± 17.80	79.17 ± 21.78	0.056	0.48	

*Note:* Values are presented as mean ± standard deviation (SD).

Abbreviations: AOU, amount of use; CR, control rehabilitation group; DAOT, digital action observation therapy group; DMT, digital mirror therapy group; MAL, Motor Activity Log; QOM, quality of movement; rNSA, Revised Nottingham Sensory Assessment; VAS, visual analogue scale.

^†^One data value was removed due to the participant answering by guessing (*n* = 5).

⁣^∗^*p* < 0.05.

**Table 4:** Between-group comparisons of the change scores on outcome measures from preintervention to postintervention (T1-T0) and from preintervention to 1-month follow-up after intervention (T2-T0).

**Outcomes**	**T1–T0**					**T2–T0**				
	**DMT** **(** **n** = 7**)**	**DAOT** **(** **n** = 6**)**	**CR** **(** **n** = 6**)**	**p** ** value**	**ε** ^2^	**DMT** **(** **n** = 7**)**	**DAOT** **(** **n** = 6**)**	**CR** **(** **n** = 6**)**	**p** ** value**	**ε** ^2^
*Primary outcome*										
FMA-UE	5.86 ± 5.84	4.83 ± 2.56	6.67 ± 4.08	0.658	0.04	7.86 ± 5.34	6.83 ± 1.94	4.83 ± 3.66	0.514	0.10
CAHAI	7.00 ± 4.97	7.67 ± 10.65	10.67 ± 5.50	0.522	0.09	10.14 ± 6.72	7.67 ± 10.17	8.50 ± 4.89	0.834	0.01
*Secondary outcome*										
rNSA–tactile sensation	3.43 ± 8.34	4.17 ± 5.57	3.60 ± 8.05^†^	0.538	0.09	10.57 ± 11.89	8.17 ± 9.54	4.00 ± 10.70^†^	0.324	0.30
rNSA–proprioception	0.86 ± 1.86	1.17 ± 1.33	0.20 ± 0.45^†^	0.569	0.07	0.14 ± 2.41	1.50 ± 1.38	0.60 ± 1.52^†^	0.407	0.19
rNSA–stereognosis	−1.71 ± 3.15	0.67 ± 2.73	1.20 ± 1.79†	0.175	0.71	−0.86 ± 3.02	1.00 ± 3.52	2.00 ± 2.83^†^	0.363	0.24
MAL–AOU	0.34 ± 0.60	0.09 ± 0.32	0.28 ± 0.33	0.374	0.22	0.31 ± 0.53	0.35 ± 0.55	0.76 ± 0.63	0.444	0.15
MAL–QOM	0.30 ± 0.44	0.20 ± 0.47	0.14 ± 0.34	0.791	0.01	0.31 ± 0.43	0.47 ± 0.60	0.52 ± 0.64	0.722	0.02
EQ-5D-5L–VAS	6.00 ± 10.97	3.33 ± 11.26	12.50 ± 14.41	0.168	0.71	8.71 ± 10.19	7.83 ± 16.68	8.33 ± 22.95	0.770	0.02

*Note:* Values are presented as mean ± standard deviation (SD).

Abbreviations: AOU, amount of use; CAHAI, Chedoke Arm and Hand Activity Inventory; CR, control rehabilitation group; DAOT, digital action observation therapy group; DMT, digital mirror therapy group; FMA-UE: Fugl-Meyer Assessment of the Upper Extremity; MAL, Motor Activity Log; QOM, quality of movement; rNSA, Revised Nottingham Sensory Assessment; VAS, visual analogue scale.

^†^One data value was removed due to the participant answering by guessing (*n* = 5).

## Data Availability

The data that support the findings of this study are available from the corresponding author on reasonable request.
